# Mechanisms of AXL overexpression and function in Imatinib-resistant chronic myeloid leukemia cells

**DOI:** 10.18632/oncotarget.360

**Published:** 2011-11-30

**Authors:** Maeva Dufies, Arnaud Jacquel, Nathalie Belhacene, Guillaume Robert, Thomas Cluzeau, Fréderic Luciano, Jill Patrice Cassuto, Sophie Raynaud, Patrick Auberger

**Affiliations:** ^1^ INSERM U895, Centre Méditerranéen de Médecine Moléculaire, Team «Cell Death, Differentiation, Inflammation and Cancer», Nice, France; ^2^ Université de Nice Sophia Antipolis, Faculté de Médecine, Nice, France; ^3^ Equipe labellisée par la Ligue Nationale Contre le Cancer 2011-2013, Paris, France; ^4^ Service d'Hématologie Clinique et de Transplantation, Nice, France; ^5^ Service d'Oncohématologie, Nice France

**Keywords:** CML, Imatinib resistance, AXL, ERK1/2, PKC

## Abstract

AXL is a receptor tyrosine kinase of the TAM family, the function of which is poorly understood. We previously identified AXL overexpression in Imatinib (IM)-resistant CML cell lines and patients. The present study was conducted to investigate the role of AXL and the mechanisms underlying AXL overexpression in Tyrosine Kinase Inhibitor (TKI)-resistant CML cells. We present evidence that high AXL expression level is a feature of TKI-resistant CML cells and knockdown of AXL sensitized TKI-resistant cells to IM. In addition, expression of wild-type AXL but not a dominant negative form of AXL confers IM-sensitive CML cells the capacity to resist IM effect. AXL overexpression required PKCα and β and constitutive activation of ERK1/2. Accordingly, GF109203X a PKC inhibitor, U0126 a MEK1 inhibitor and PKCα/β knockdown restore sensitivity to IM while PKCα or PKCβ overexpression in CML cells promotes protection against IM-induced cell death. Finally, using luciferase promoter activity assays we established that AXL is regulated transcriptionally through the AP1 transcription factor. Our findings reveal an unexpected role of AXL in resistance to TKI in CML cells, identify the molecular mechanisms involved in its overexpression and support the notion that AXL is a new marker of resistance to TKI in CML.

## INTRODUCTION

A feature of chronic myeloid leukemia (CML) cells is the presence of the Philadelphia chromosome (Ph) that results from the t(9;22)(q34;q11) translocation [1]. The Ph gene encodes BCR-ABL, an oncogenic fusion protein. In CML, p210-BCR-ABL is a constitutively activated tyrosine kinase that triggers several signaling pathways which collectively confers CML cells a proliferative advantage and resistance to apoptosis [2-5]. Imatinib (IM), a tyrosine kinase inhibitor (TKI) is used frequently as the first line treatment for patients suffering CML [6, 7], even though other TKIs including Dasatinib and Nilotinib are now also given as a first line therapy in CML patients. Nevertheless, resistance to TKI which occurs in all phases of the disease [8] represents a recurrent problem for CML patients. Importantly, activation of tyrosine kinases including LYN, FYN, and AXL has been reported in IM and Nilotinib-resistant cell lines and also in some CML patients treated with such inhibitors [9-11]. Therefore, targeting tyrosine kinases, expression of which is elevated in resistant patients could be a pertinent option to treat TKI-refractory patients.

We have shown previously that AXL is overexpressed in CML cell lines resistant to IM and PD166326, a dual BCR-ABL and SRC kinase inhibitor of the Dasatinib family. AXL is a member of the TAM family of receptor tyrosine kinase (RTK) initially isolated from primary human myeloid leukemia cells [12]. The TAM receptor family also includes the Tyro-3 and Mer receptors which share with AXL subsequent sequence homologies [13]. AXL overexpression is a common feature of cancer cells. Moreover, increased AXL level and activation have been linked with Imatinib resistance in gastrointestinal stromal tumors (GIST) [14], Nilotinib resistance in CML cells [11], resistance to BMS-754087 in Rhabdomyosarcoma [15], Lapatinib resistance in HER-2 positive breast tumor cells [16] and in Cisplatin-resistant ovarian cancer [17]. AXL has been also shown to be induced by chemotherapy drugs in acute myeloid leukemia [18] and AXL is consistently associated to drug-mediated resistance in several types of cancer [15, 16, 18, 19]. Although, it seems well established that AXL is involved in resistance to chemotherapy in cancer cells, the mechanisms underlying AXL overexpression in this context remain unexplored.

In the present study, we report compelling evidence that AXL is overexpressed in several TKI-resistant CML cell lines. Importantly, we establish that AXL knockdown using specific siRNA sensitized TKI-resistant cells to IM effect. Moreover, overexpression of AXL-WT but not AXL-DN which lacks the kinase domain allowed IM-sensitive CML cells to resist IM treatment. Importantly, we also bring evidence that PKCα and β and constitutive activation of the ERK1/2 pathway are required for AXL overexpression in TKI-resistant cells lines. Accordingly, pharmacological inhibition of classical PKC and MEK1 or PKCα/β silencing by specific siRNA restores sensitivity to Imatinib while PKCα and PKCβ overexpression or PMA treatment in TKI-sensitive cells confers protection against Imatinib-induced cell death. In addition, luciferase promoter activity assays also indicate that AXL overexpression is regulated at the transcriptional level by the AP1 transcription factor in TKI-resistant cells. Collectively, our findings demonstrate the role of AXL in resistance to TKI in CML cells and identify the involvement of PKC and ERK1/2 pathways in AXL accumulation. Our data support the notion that AXL could be a pertinent prognostic marker for resistance to TKI in CML cells.

## RESULTS

### AXL overexpression protects IM-R and PD-R cells against IM effects

**Figure 1:**
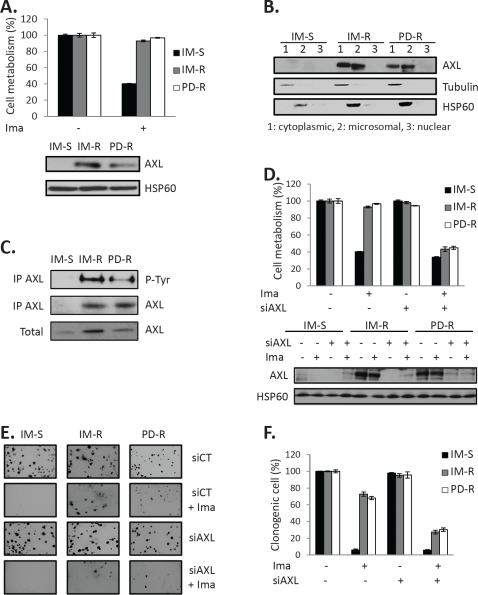
AXL is overexpressed in TKI-resistant CML cell lines and protects them against TKI-induced apoptosis ***(A)*** IM-S, IM-R and PD-R cells were incubated for 48h with 1μM IM. Cell metabolism was measured using the XTT assay (top panel). Protein extracts were prepared, and 50μg of proteins was subjected to SDS-PAGE followed by immunoblot analysis (bottom panel). ***(B)*** Cell extracts were separated into cytosol, mitochondria and nuclear-enriched fractions and AXL location was assessed by Western blot in the different fractions. HSP60 and tubulin served as both loading control and validation of the membrane and cytoplasmic fractions, respectively. ***(C)*** Cell extracts were prepared and AXL was immuno-precipitated with an anti-AXL antibody. AXL phosphorylation was analyzed by western blot using the 4G10 anti-phosphotyrosine antibody. ***(D)*** Cells were transfected with control or AXL siRNAs. 48h after transfection, cells were treated with IM (1μM) for 48h. Cell metabolism was measured using the XTT assay and AXL expression was analyzed by Western blot. ***(E and F)*** Cells were transfected with control or AXL siRNAs. After 48h, IM (1μM) was added to cell lines growing in semi-solid methylcellulose medium ***(E)***. ***(F)*** Results are expressed as the percentage of colony forming cells after drug treatment in comparison with the untreated control cells.

We have previously reported that AXL is overexpressed in IM-resistant CML cell lines [9, 20]. This was confirmed in K562 cells resistant to the dual SRC/BCR-ABL inhibitor PD166326 (PD-R) cells (Figure 1A). AXL was present in both the cytoplasmic and microsomal fractions of TKI-resistant cell lines, but was absent from the nuclear fraction (Figure 1B). To assess the level of phosphorylation of AXL, we performed immuno-precipitation assays from IM-S, IM-R and PD-R CML cells followed by western blot with the specific 4G10 anti-phosphotyrosine monoclonal antibody (Figure 1C). As expected, AXL phosphorylation, which reflected AXL activity, was found only in IM-R and PD-R resistant cell lines. To investigate the role of AXL overexpression in resistance to TKI in K562 CML cells we used a siRNA approach. A siRNA directed against AXL efficiently inhibited AXL expression in both IM-R and PD-R CML cells (Figure 1D, bottom panel). AXL knockdown by itself had no effect on cell metabolism but sensitized drastically IM-R and PD-R cells to IM-mediated loss of cell metabolism (Figure 1D, top panel) and also decreased total cell counts (Sup Figure 1A). As expected, IM abolished the clonogenic potential of IM-S cells but had minimal effect on IM-R and PD-R CML cells (Figure 1E). Knockdown of AXL by itself failed to affect the clonogenic potential of IM-R and PD-R but strongly reduced the number of colonies when combined with IM. Indeed, quantitative analysis of colonies counts by the Image J software revealed a 80% decrease in the number of IM-R and PD-R colonies in the presence of the combination of the AXL siRNA and IM versus only 25% with IM alone (Figure 1F). Knockdown of AXL also sensitized IM-R and PD-R cells to the effect of PD166326 (Sup Figure 1B). By contrast, AXL knockdown did not modify the effect of staurosporine on cell metabolism linking specifically AXL expression to IM resistance (Sup Figure 1C).

### Knockdown of AXL induces apoptosis of TKI-resistant CML cell lines

**Figure 2:**
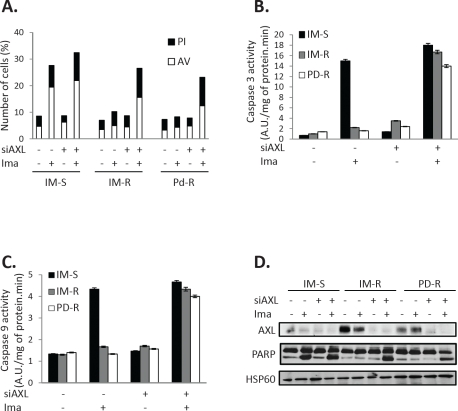
AXL extinction sensitizes TKI-resistant CML cells to IM Cells were transfected with control or AXL siRNA. 48h after siRNA transfection cells were treated with IM (1μM) for 48h. ***(A)*** Cells were stained with the PI/ annexin-V-fluos staining kit according to the manufacturer's indications. Histograms show both annexin-V^+^/PI^−^ cells (open bars) and annexin-V^+^/PI^+^ cells (filled bars). ***(B, C)*** Cells were lysed in caspase buffer and caspase-3 ***(B)*** and -9 activities ***(C)*** were evaluated in quadruplicate using 0.2mM Ac-DEVD-AMC or Ac-LEDH-AMC as substrates. Results expressed as arbitrary units (A.U.) /mg of protein.min and are means ± Standard deviation of 4 independent experiments made in quadruplicate. Error bars 95% confidence intervals. ***(D)*** AXL expression and cleavage of PARP was analyzed by Western blot.

To determine whether loss of cell metabolism and clonogenic potential reflected increased cell death, IM-S, IM-R and PD-R CML cell lines were treated or not with IM in the presence or the absence of a siRNA directed against AXL. As expected IM increased the number of dead cells from 8 to 28% but had no effect on both IM-R and PD-R cells (Figure 2A). AXL knockdown restored sensitivity to IM in both IM-R and PD-R cells to almost the same level than in control cells (26 and 22% dead cells respectively versus 33% in IM-S cells). Accordingly, AXL knockdown specifically increased caspase 3 and 9 activities and Poly-ADP-Ribose-Polymerase cleavage (Figures 2B, C and D). To verify that the observed effects were not due to clonal variations, we also analyzed the IM-resistant cell line JURLMK1-R. Resistance of JURLMK1-R to IM was also correlated with overexpression of AXL (Sup Figure 2A). Identical results were obtained regarding cell metabolism when JURLMK1-R CML cells were assessed for annexin V/PI staining and caspase 3 activity (Sup Figures 2B, C and D). All together our findings show that increased cell death is one the main mechanisms by which the AXL siRNA exerted its effect. Moreover, our data also highlight that AXL overexpression and/or activity could represent a hallmark of resistance to TKI in CML (8, 11). Finally, the importance of caspases in the pro-apoptotic effect of the AXL siRNA was confirmed using the pan caspase inhibitor z-VAD-fmk which was found to block the effect of AXL silencing on both cell metabolism and caspase 3 activation (Sup Figure 3A and B).

### Characterization of the mechanisms involved in AXL overexpression

To investigate the possible mechanisms involved in AXL overexpression in IM-R and PD-R cells we first used a pharmacological approach. Among the different compounds used, only GF109203X (GFX), an inhibitor of classical and new PKCs, and U0126, a MEK1 inhibitor, dampened AXL expression in IM-R and PD-R CML cell lines (data not shown). Therefore both PKC and MEK/ERK pathways are involved in AXL overexpression in TKI-resistant CML cell lines. If GFX was capable to inhibit AXL expression levels in IM-R and PD-R CML cells, it should sensitize these cells to IM effect. Although GFX by itself induced only a limited loss of cell metabolism in TKI-resistant cells, it synergized with IM to promote loss of cell metabolism in IM-R and PD-R cells (Sup Figure 4A and B). Accordingly, GFX also sensitized IM-R and PD-R CML cells to IM-induced apoptosis as judged by increased annexin V staining (Sup Figure 4C) and caspase 3 activity (Sup Figure 4D).

### PKCα and β are responsible for increased AXL expression in resistant CML cells

**Figure 3:**
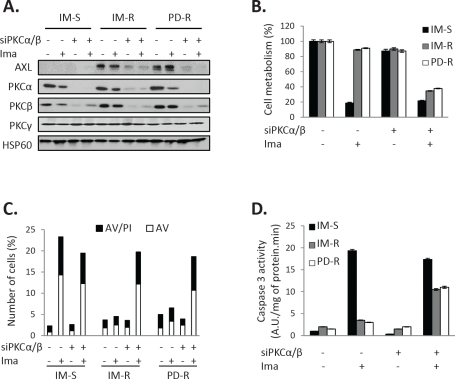
Inhibition of PKCα/β signaling sensitizes TKI-resistant cells to IM-induced apoptosis Cells were transfected with control siRNA or a dual PKCα/β siRNA. 48h after siRNA transfection cells were treated with IM (1μM) for 48 h. ***(A)*** PKCα, β and γ expression was analyzed by western blot. ***(B)*** Cell metabolism was measured using the XTT assay. ***(C)*** Cells were stained with the PI/annexin-V-fluos staining kit and analyzed as described in Fig 2A. ***(D)*** Caspase 3 activity was assessed as described in Fig 2B.

**Figure 4:**
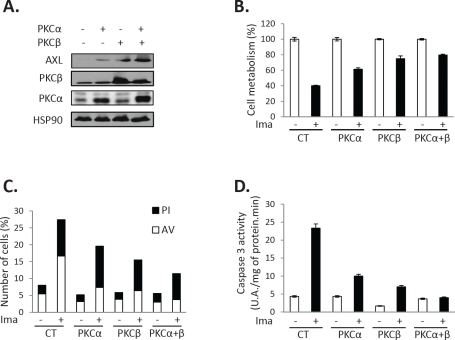
Overexpression of PKCα/β in IM-S CML cells confers protection against apoptosis PKCα and/or PKCβ plasmids were transfected in IM-S cells. 48h after transfection cells were treated with IM (1 μM) for 48 h. ***(A)*** AXL, PKCα or β expression was analyzed by western blot. ***(B)*** Cell metabolism was measured using the XTT assay. ***(C)*** Cells were stained with the PI/annexin-V-fluos staining kit as described in Fig 2A. ***(D)*** Caspase 3 activity was assessed as described in Fig 2B.

As GFX drastically inhibits AXL expression in both IM-R and PD-R cells, we postulated that classical and/or new PKC isoforms could be responsible for increased expression of this RTK. We have reported previously that PKCα and β are strongly expressed in K562 cells [21, 22]. Importantly, abrogation of both PKCα and β expression by a dual PKCα/β siRNA results in a parallel decrease of AXL expression, irrespectively of the presence or the absence of IM (Figure 3A). Knockdown of PKCα/β was accompanied by a sensitization to IM treatment in accordance with the effect of pharmacological inhibition of PKCs (Figure 3B and Sup Figure 5B). Importantly, silencing of both PKCα and β led to an increase in Annexin V staining (Figure 3C) and caspase 3 activity (Figure 3D) only in TKI-resistant cells treated with Imatinib. In addition, overexpression of PKCα or β induced AXL expression in IM-S K562 cells, while co-expression of both isoforms triggered further AXL accumulation (Figure 4A). PKCα and β were also shown to protect IM-S cells from IM-mediated loss of cell metabolism (Figure 4B) and apoptosis as judged by AnnexinV/PI staining (Figure 4C) and caspase 3 activity (Figure 4D), even though co-transfection of both isoforms conferred further protection.

### Overexpression of AXL-WT or AXL-DN differently affects IM-mediated apoptosis

**Figure 5:**
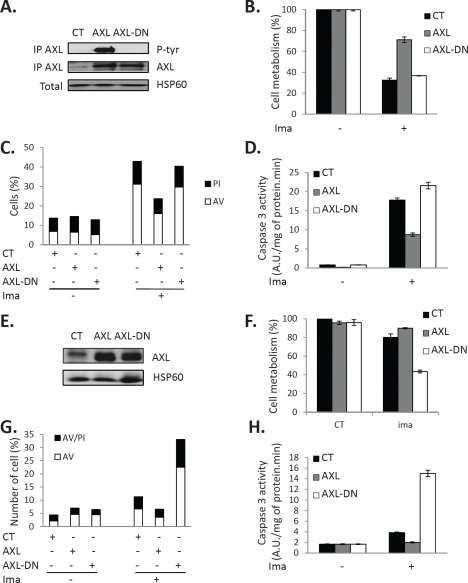
Over-expression of AXL renders IM-S cells resistant to IM AXL-WT or AXL-DN plasmids were transfected in IM-S ***(A-D)*** or IM-R cells ***(E-H)***, respectively. 48 h after transfection, cells were treated with IM (1μM) for 48h. ***(A and E)***, cells were lysed, immuno-precipitated with an AXL antibody and phosphorylation of AXL analyzed by Western blot using a phospho-tyrosine antibody (4G10). ***(B and F)*** Cell metabolism was measured using the XTT assay. ***(C and G)*** Cells were stained with the PI/annexin-V-fluos staining kit for evaluation of dead and apoptotic cells. ***(D and H)*** Caspase 3 activity was assessed as described in Fig 2.

As previously mentioned, PKCα and β can protect cells from IM-induced cell death by different mechanisms including AXL overexpression. To assess the implication of AXL in CML cell protection, we next overexpressed a wild-type (AXL-WT) or a kinase deleted mutant of AXL (AXL-DN) in IM-S K562 cells. We first checked expression and phosphorylation of AXL in transfected cells. As shown on Figure 5 A, both forms of AXL protein were present at a similar level in IM-S cells, but tyrosine phosphorylation of AXL was detected only in cells transfected with the wild-type construct. Importantly, AXL-WT but not AXL-DN over-expression conferred IM-S cells protection against IM-induced loss of cell metabolism (Figure 5B), apoptosis as judged by Annexin V/PI staining (Figure 5C) and caspase 3 activity (Figure 5D). Importantly, no protective effect was observed with AXL-DN indicating that the protective effect of AXL was strictly dependent on its kinase activity. The protection triggered by AXL overexpression although not complete was equivalent to the one induced by PKCα or β (Figure 4) suggesting that AXL is one if not the main cause of resistance to IM in IM-R and PD-R CML cell lines. When the same experiments were reproduced in IM-R cells, increased level of AXL-WT and AXL-DN were detected (Figure 5E). Overexpression of AXL-WT in IM-R cells failed to alter cell metabolism, whereas AXL-DN inhibited drastically cell metabolism in the presence of IM (Figure 5F). This was also correlated with increased cell death as assessed by Annexin V/PI staining (Figure 5G) and caspase 3 assay (Figure 5H).

### Role of ERK1/2 activation in AXL overexpression

**Figure 6:**
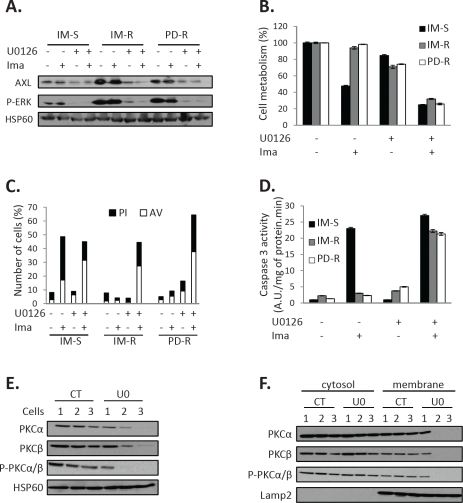
U0126 sensitizes TKI-resistant CML cell lines to IM via inhibition of PKCα and β translocation ***(A-D)*** Cells were treated with U0126 (10μM). 48h after, cells were treated with Imatinib (1μM) for 48h. ***(A)*** AXL, ERK expression and Phospho-ERK status were analyzed by Western blot. ***(B)*** Cell metabolism was measured using the XTT assay. ***(C)*** Cells were stained with the PI/annexin-V-fluos staining kit and analyzed as described in Figure 2A. ***(D)*** Caspase 3 activity was assessed as described in Fig 2B. ***(E-F)*** Cells were treated with U0126 (10μM) for 4h. ***(E)*** PKCα, PKCβ expression and Phospho-PKCα/β status were analyzed by Western blot. ***(F)*** Cell extracts were separated into cytosol and membrane fractions and PKCα, PKCβ and Phospho-PKCα/β location was assessed by Western blot in the different fractions. Lamp2 served as loading control and validation of the microsomal fraction.

We reported earlier that ERK1/2 is constitutively activated in IM-R CML cells [20]. Accordingly, AXL overexpression correlated also with persistent activation of ERK1/2 in IM-R and PD-R CML cells (Figure 6A). Interestingly, inhibition of ERK1/2 by the MEK1 inhibitor U0126 abolished AXL expression (Figure 6A). In addition U0126 also sensitized TKI-resistant CML cells to IM (Figure 6B). Finally, U0126 increased both IM-induced annexin V/PI staining (Figure 6C) and caspase 3 activity (Figure 6D) in IM-R and PD-R CML cell lines. Taken together our data established that ERK1/2 is required for AXL expression upstream of PKCα and β in TKI-resistant CML cell lines. In addition, Figure 6E showed that IM-S, IM-R and PD-R cell lines expressed the same level of PKCα and β expression and phosphorylation. Importantly, U0126 significantly inhibited expression of PKCα and β in IM-R and PD-R cells but not in their IM-sensitive counterpart. Translocation rather than global expression of both PKC isoforms was impaired in IM-R cells as judged by subcellular fractionation (Figure 6F). Collectively, our findings support the notion that in IM-resistant CML cells constitutive activation of the ERK pathway is responsible for PKCα and β activation.

### AP1 is required for AXL overexpression in TKI-resistant CML cells

**Figure 7:**
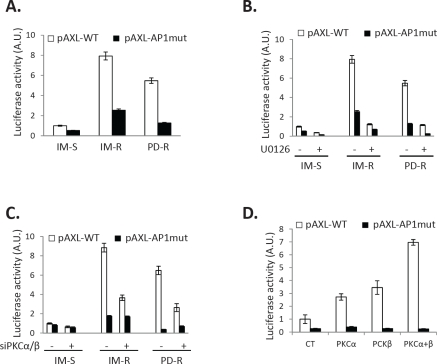
Transcriptional regulation of AXL in TKI-resistant CML cells Cells were transfected with luciferase reporter plasmids (pAXL-WT or pAXL-DN muted). ***(A)*** 24h after transfection, cells were lysed and luciferase activity was measured to determine AXL promoter activity. ***(B, D)*** 24 h after transfection, cells were treated with 10μM U0126 for 24h ***(B)***. Cells were then lysed and luciferase activity was measured. ***(C, D)*** 24 h after transfection, cells were transfected with a dual PKC α/β siRNA ***(C)*** or with PKC α or/and PKC β plasmid ***(D)*** for 24h. Cells were then lysed and luciferase activity measured. ***(A to E)*** Results are expressed as arbitrary units (A.U.) / mg of protein. min and are means ± standard deviation of 3 independent experiments made in triplicate. Error bars 95% confidence intervals.

To get insights into the mechanisms of AXL regulation in CML cells we performed luciferase activity assays using a minimal AXL promoter carrying or not a mutation on its AP1 binding site. Promoter activity was 8 and 6 times higher in IM-R and PD-R cells respectively compared to control cells, reflecting increased AXL transcription in these cell lines (Figure 7A). When using the mutated promoter there was an 80% decrease in luciferase activity arguing for an important role of AP1 in AXL overexpression. AXL transcription was abolished by U0126 highlighting the role of ERK1/2 in this process (Figure 7B). Accordingly, knockdown of PKCα/β was associated with a drastic inhibition of AXL transcription in IM-R and PD-R CML cells (Figure 7C). Finally, overexpression of PKCα or PKCβ triggered activation of the AXL promoter, the combination of both being more efficient that the individual expression of one of these isoforms (Figure 7D). Finally, it is worth noting that the level of activation of the AXL promoter was identical whatever the situation in TKI-resistant cells and IM-S cells transfected with the combination of PKCα and β, highlighting the role of PKCs in the regulation of AXL.

## DISCUSSION

We report here that over-expression of the RTK AXL is consistently detected in several TKI-resistant CML cell lines and that silencing of AXL with specific siRNA sensitizes TKI-resistant cells to IM-mediated loss of cell viability and apoptosis. We also bring evidence that a WT, but not a dominant-negative AXL protein lacking the kinase domain, confers IM-sensitive cells the capacity to resist IM treatment. Pharmacological inhibition of classical PKC and MEK1 or PKCα/β silencing by specific siRNA restores sensitivity to IM while PKCα and PKCβ overexpression in TKI-sensitive cells confers protection against IM-induced cell death demonstrating that both PKCα and β and constitutive activation of ERK1/2 are required for AXL overexpression. In addition, we show that AXL overexpression is regulated at the transcriptional level by the AP1 transcription factor in TKI-resistant cells.

### Increased expression of AXL is involved in resistance to TKI

Increased expression of AXL is a recurrent characteristic of cancer cells. AXL overexpression and/or activation has been linked with resistance to chemotherapy in gastrointestinal stromal tumor cell lines [14], in CML cells [11], rhabdomyosarcoma [15], HER-2 positive breast tumor cells [16] and ovarian cancer [17]. In addition, AXL has been shown to be induced by chemotherapy drugs in acute myeloid leukemia [18]. Accordingly, a recent study has reported that inhibition of AXL expression in Nilotinib-resistant CML cells lines can restore sensitivity to Nilotinib [11]. Indeed, Gioia et al. showed that AXL interacts with both Syk and Lyn to mediate resistance to Nilotinib in the K562 CML cell line. Interestingly, these Nilotinib-resistant CML cells were shown to display constitutive activation of Lyn a situation that is closed to the one observed in our TKI-resistant K562 cell lines where the Fyn but not Lyn tyrosine kinase is constitutively activated and participates to persistent ERK1/2 activation [9, 10, 20]. Therefore, AXL activation in TKI-resistant CML cells could be achieved by two different but complementary pathways, increased signaling or increased protein expression, both leading to resistance to TKIs. It has been reported that dominant-negative inhibition of AXL suppresses brain tumor growth and invasion and prolongs survival in a mice model of gliomagenesis [23]. This was the first direct proof that the tyrosine kinase activity of AXL can be involved in tumor progression. Accordingly, we established here using AXL-DN constructs that AXL activity is necessary to confer resistance to IM-sensitive cells.

### Regulation of AXL expression in TKI resistant cell lines

It is well established that AXL stimulated cell proliferation through the MAPK/ERK and PI3K/AKT pathways [19, 24-26]. As mentioned before, we have shown previously that IM-R and PD-R CML cells exhibited persistent activation of a Fyn/ERK module that participates to TKI-resistance [20]. However, overexpression of AXL in TKI-resistant cells is not responsible for increased ERK activity since knockdown of AXL using specific siRNA failed to inhibit constitutive ERK activation in IM-R and PD-R cells (data not shown). Caveat to that, AXL overexpression is dependent on persistent ERK activation since inhibition of ERK by U0126 blocked AXL transcription and expression in IM-R and PD-R CML cells leading to increased apoptosis and caspase activation. Besides ERK, we bring for the first time evidence that PKCα and β are also involved in AXL overexpression. This observation is in good agreement with the fact that AXL has been identified initially as a gene induced by phorbol esters in CML cells [21, 27, 28].

### AXL expression is regulated at the transcriptional level in resistant CML cells

The mechanisms governing AXL expression are poorly understood. However, in a recent study, it has been reported that PMA-mediated increased in AXL is regulated at the transcriptional level essentially through the AP1 transcription factor [27]. Accordingly, using luciferase assays, we established that PKCα and β were required for increased transcription of AXL in IM-R cells. Importantly, pharmacological and siRNA approaches allowed us to determine that increased transcription and expression of AXL in TKI-resistant cells are dependent on both ERK and PKCα and β signaling. One important question that remains to be answered is whether ERK1/2 and PKC act independently to promote AXL accumulation in TKI-resistant or whether it exist a hierarchy of activation of these kinase pathways. Importantly, we established the hierarchy of kinase activation leading to AXL overexpression. Indeed, we showed that ERK1/2 is involved upstream of PKCα and β to regulate AXL expression in TKI-resistant CML cells since inhibition of MEK1 by U0126 abrogated PKCα and β translocation and as consequence on AXL expression only in IM-resistant cells. Collectively our finding supports the notion that the constitutive activation of ERK in TKI-resistant cells control translocation and activity of PKCα and β which in turn are involved in increased transcription and expression of AXL.

### Targeting AXL in TKI-resistant CML cell lines

Findings from other and our present data clearly indicate that increased expression of AXL is a common feature of cancer cells [26, 29-32]. This seems particularly true regarding CML and AML. Moreover, increased expression of AXL is often associated to chemotherapy drug resistance [15, 16, 18, 19]. Therefore, targeting AXL could represent a promising strategy for TKI-resistant patients. Until now, no specific inhibitors of AXL have been characterized. MP470 is reported to inhibit AXL *in vitro* and *in vivo* [14] but is also effective at identical concentrations on other RTKs [33, 34]. For instance, MP470 was found effective to sensitize the IM-resistant GIST882 cell line to IM, but it is not known from the results of this study whether this effect is due to effective AXL inhibition. In this line, we have shown that MP470 efficiently killed IM-R K562 cells. However, MP470 was found to affect IM-S K562 cell as well, strongly suggesting that at least in the case of CML, AXL is not an MP470 target. In the absence of highly specific AXL inhibitor the use of siRNA directed against AXL have however brought clear evidence that AXL is involved in TKI-resistance. The development of highly specific inhibitors of AXL will certainly help deciphering further the role of this RTK in resistance to TKI and to propose new therapeutic strategies in hematopoietic malignancies [35].

## Materials and Methods

### Reagents and antibodies

Imatinib mesylate (STI571, Gleevec) was purchased from Enzo Life Sciences (Farmingdale, NY, USA) and RPMI 1640 medium, IMDM medium and fetal calf serum (FCS) from life technologies (Carlsbad, CA, USA). Sodium fluoride, sodium orthovanadate, phenyl-methyl-sulfonyl fluoride (PMSF), aprotinin, leupeptin and Phorbol-12-Myristate-13-Acetate (PMA) were purchased from Sigma-Aldrich (France). Ac-DEVD-7-amino-4-methylcoumarin (AMC), Ac-LEHD-AMC, Ac-DEVD-CHO, Ac-LEHD-CHO and zVAD-fmk were from peptanova (PeptaNova GmbH, Sandhausen, Germany). Anti-HSP90, anti-HSP60, anti-tubulin and anti-AXL antibodies were purchased from Santa Cruz Biotechnology (Santa Cruz, CA, USA). HRP conjugated anti-mouse and anti-goat antibodies were from Dakopatts (Glostrup, Denmark). Anti-phosphotyrosine (P-Tyr), anti-PARP, anti-PKCα, anti-PKCβ, anti-PKCγ, anti-phospho-ERK, and peroxydase-conjugated anti-rabbit antibodies were purchased from Cell Signaling Technology (Beverly, MA, USA). Staurosporine, U0126, SP600125, SU6656, GF109203X, PP2 and SB202474 were from Calbiochem (San Diego, USA).

### RNA interference

AXL (UCGCGGUGCUGGGAGCUAA) and PKCα/β (life technologies) specific siRNAs from Life Technologies were transfected in cells using Lipofectamine RNAiMAX reagent as described previously [36].

### Plasmids and luciferase reporter constructs

AXL-WT and AXL-DN (kinase-deleted) plasmids have been published elsewhere [23] and were kindly provided by Pr Axel Ullrich. PKCα and PKCβ have been described previously [22]. Luciferase reporter constructs of the AXL promoter were kindly provided by Dr Heile Allgayer. pAXL-WT represents the WT luciferase reporter construct (−1276/+7) and pAXL-AP1mut corresponds to luciferase reporter construct mutated into position (−637 to −650 AP1 corresponding to the AP1 binding site) [27].

### Cell lines

The human CML cells lines IM-S (K562) and JURLMK1 were grown at 37°C under 5% CO2 in RPMI supplemented with 5% FCS, 50 U/ml penicillin, 50 μg/ml streptomycin, and 1 mM sodium pyruvate. Establishment of resistant K562 cell clones (IM-R and PD-R) and resistant JURLMK1 cell clones (JURLMK1-R) have been described previously [9].

### Colony formation assay

IM (1μM) was added to cells lines growing in semisolid methylcellulose medium (0.5x10^3^ cells/ml; MethoCult H4236; StemCell Technologies Inc, Vancouver, BC, Canada). Colonies were detected after 10 days of culture by adding 1 mg/ml of 3-(4,5-dimethylthiazol- 2-yl)-2,5-diphenyltetrazolium bromide (MTT) reagent and were scored by Image J quantification software (U.S. National Institutes of Health, Bethesda, MD, USA) [37].

### Caspase assays

Caspase assays have been described in details elsewhere [4].

### Flow cytometry

*Apoptosis analysis:* After stimulation, cells were washed with ice-cold PBS and were stained with the annexin-V-fluos staining kit (Roche, Meylan, France) according to the manufacturer's procedure. Fluorescence was measured by using the FL2 channels of a fluorescence-activated cell sorter apparatus (Miltenyi cytometer).

### Western blot

After stimulation, cells were harvested and lysed in buffer containing 1% Triton X-100 and supplemented with protease and phosphatase inhibitors (Roche Diagnostics). Lysates were pelleted, and 50 μg of protein were analyzed by SDS-PAGE as described previously [38].

### Preparation of cytoplasmic, microsomal and nuclear fractions

After stimulation, cells were washed and lysed as described previously [36]. Proteins contained in cytosolic, microsomal and nuclear fractions were separated by SDS-PAGE and transferred onto PVDF membrane before incubation with specific antibodies.

### Cell viability (XTT)

Cells (15x10^3^ cells/100 μl) were incubated in a 96-well plate with different effectors for the times indicated in the figure legends. Fifty microliters of sodium 3′-[1-phenylaminocarbonyl)-3,4-tetrazolium]-bis(4-methoxy-6-nitro) benzene sulfonic acid hydrate (XTT) reagent was added to each well. The assay is based on the cleavage of the yellow tetrazolium salt XTT to form an orange formazan dye by metabolically active cells. The absorbance of the formazan product, reflecting cell viability, was measured at 490 nm. Each assay was performed in quadruplicate.

### Transfection and luciferase assays

For luciferase assays, 10^6^ cells were plated in 24-well plates and cells were electroporated with the Amaxa Cell Line Nucleofector Kit V (Lonza, Switzerland) with 1 μg of respective reporter plasmid and other plasmid. After 16 h of transfection, cells were treated with specific inhibitors for 24 h. Then, cells were lysed with 80 μl of lysis buffer (Promega, France) for 20 min. Luciferase activity of 20 μl of cell lysate was measured via the dual-luciferase reporter assay system.

## Supplementary Figures


