# Supplementary material for: Mechanisms of AXL overexpression and function in Imatinib-resistant chronic myeloid leukemia cells
Source: Oncotarget. 2011 Nov 30;2(11):874–85. doi: 10.18632/oncotarget.360 (PMC3259992; doi:10.18632/oncotarget.360)
Supplement: Supplementary file 1 [file oncotarget-02-874-s001.pdf]

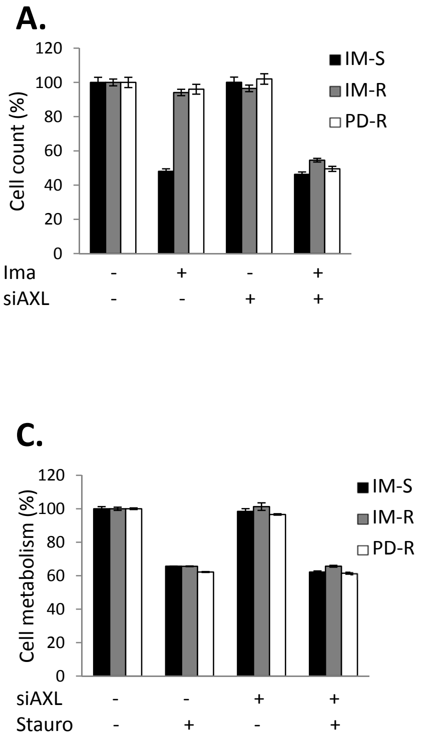

Sup Figure 1: Cells were transfected with control siRNA or AXL siRNA. ***(A)*** 48h after siRNA transfection cells were treated with staurosporine (5µM) for 48h. Cells metabolism was measured using the XTT assay. ***(B)*** 48h after siRNA transfection cells were treated with Imatinib (1µM) for 48h. Cell numbers was assessed by flow cytometry. Data are the mean of three independent experiments made in quadruplicates. ***(C)*** 48h after siRNA transfection cells were treated with PD166326 (50nM) for 48h. Cells metabolism was measured using the XTT assay.

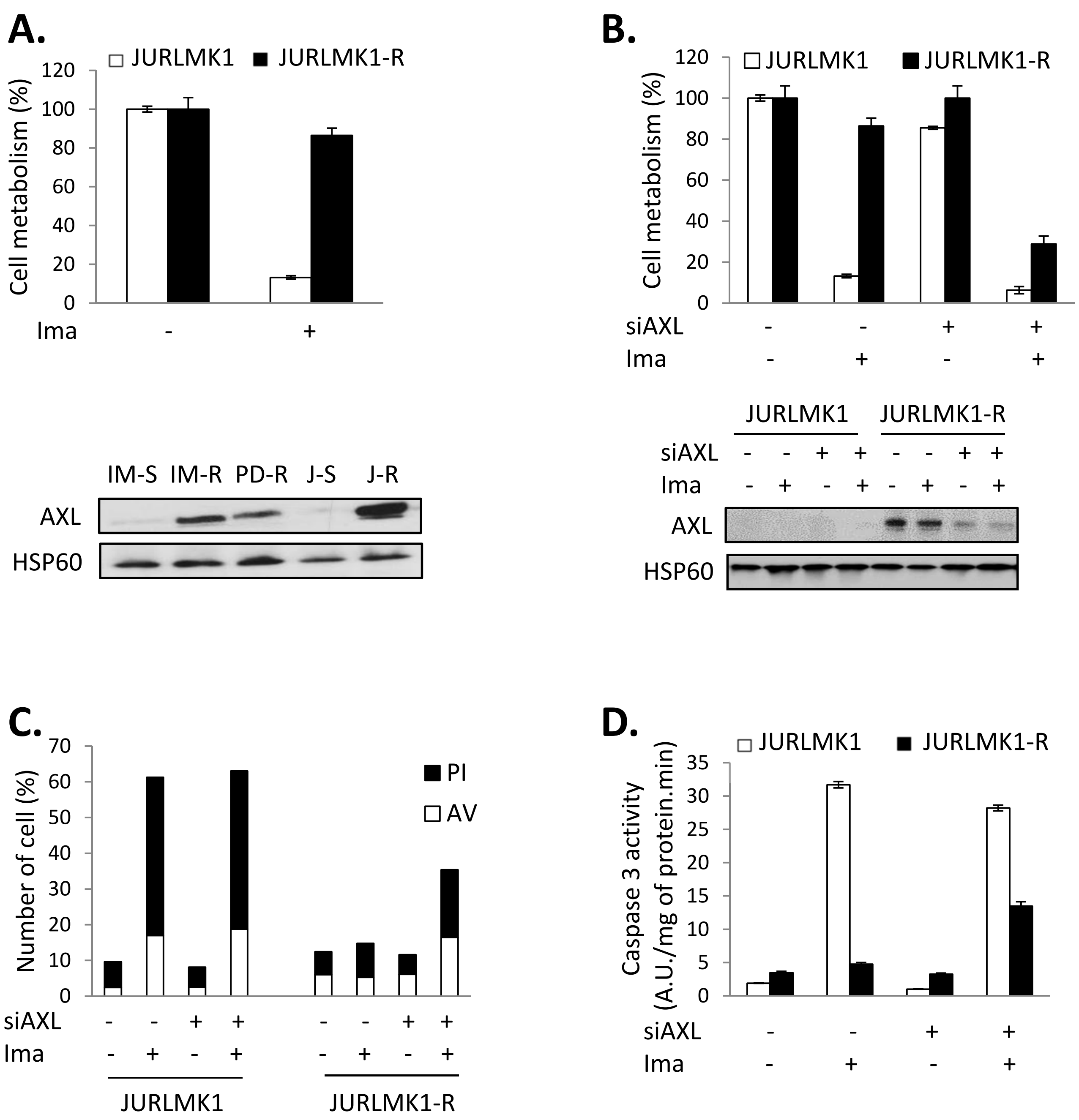

Sup Figure 2. ***(A)*** JURLMK1 and JURLMK1-R cells were incubated for 48h with 1µM of Imatinib. Cells metabolism was measured using the XTT assay. Protein extracts were prepared, and 50µg of proteins was subjected to SDS-PAGE followed by immunoblot analysis. ***(B-D)*** Cells were transfected with control siRNA or AXL siRNA. 48h after siRNA transfection cells were treated with Imatinib (1µM) for 48h. ***(B)*** Cells metabolism was measured using the XTT assay and AXL expression was analyzed by western blot. ***(C)*** Cells were stained with the PI and Annexin-V-fluos staining kit according to the manufacturer's indications. Histograms show both annexin-V^+/PI^- cells (open bars) and annexin-V^+/PI^+ cells (filled bars). ***(D)*** Caspase 3 activity was assessed as described in Fig 2B.

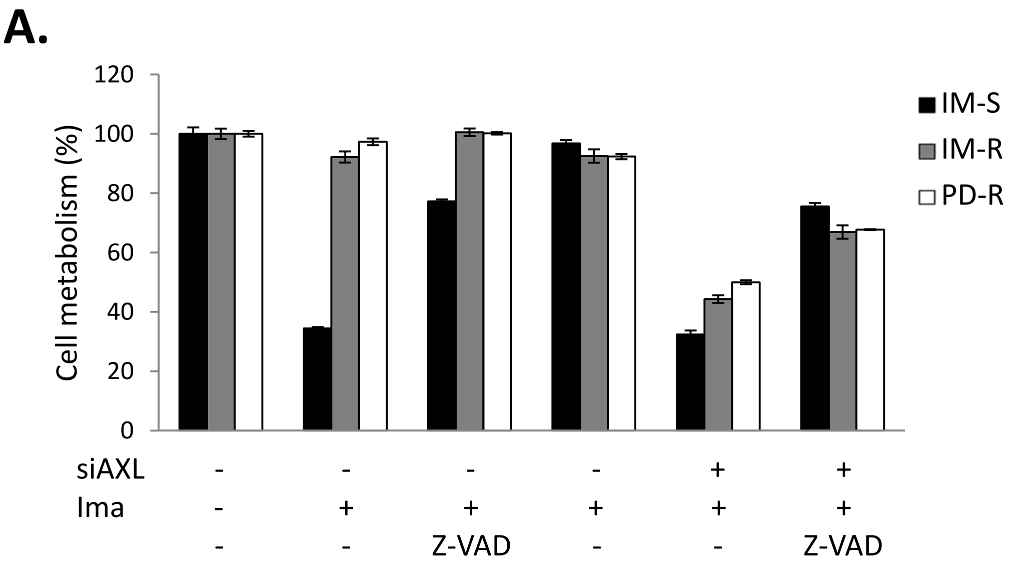

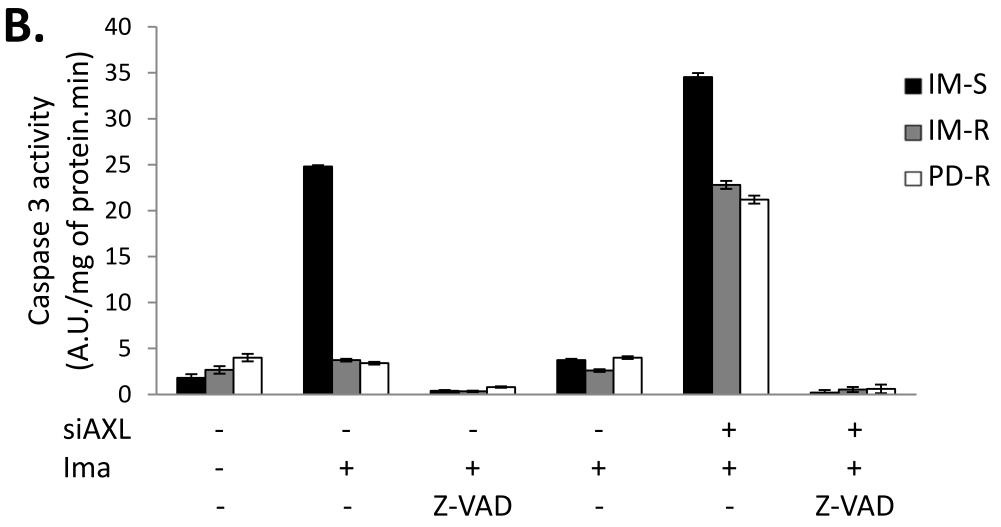

Sup Figure 3. Cells were transfected with control siRNA or AXL siRNA. 48h after siRNA transfection cells were treated with Imatinib (1µM) for 48h in presence or in absence of zVAD-fmk (50µM). ***(A)*** Cells metabolism was measured using the XTT assay. ***(B)*** Caspase 3 activity was assessed as described in Fig 2B.

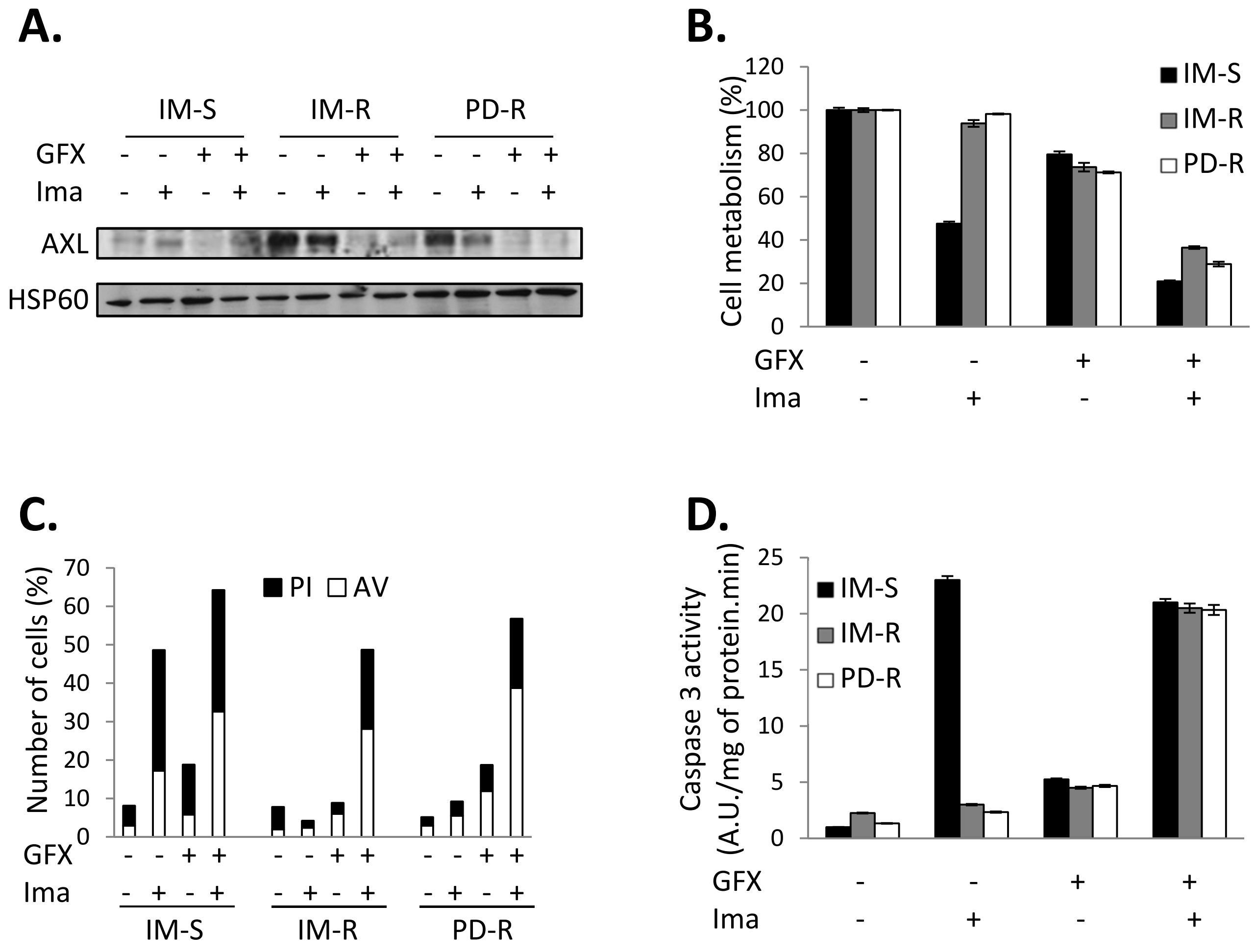

Sup Figure 4. Cells were incubated for 24h with 5µM of GF109203X (GFX) and after with 1µM of Imatinib during 48h. ***(A)*** AXL expression was analyzed by western blot. ***(B)*** Cells metabolism was measured using the XTT assay. ***(C)*** Cells were stained with the PI and annexin-V-fluos staining kit according to the manufacturer's indications. Histograms show both Annexin-V^+/PI^- cells (open bars) and annexin-V^+/PI^+ cells (filled bars). ***(D)*** And finally, caspase 3 activity was assessed as described in Fig 2B.
